# Pro-Secretory Activity and Pharmacology in Rabbits of an Aminophenyl-1,3,5-Triazine CFTR Activator for Dry Eye Disorders

**DOI:** 10.1167/iovs.17-22525

**Published:** 2017-09

**Authors:** Christian M. Felix, Sujin Lee, Marc H. Levin, Alan S. Verkman

**Affiliations:** 1 Departments of Medicine and Physiology, University of California, San Francisco, California, United States; 2 Department of Ophthalmology, Palo Alto Medical Foundation, Palo Alto, California, United States

**Keywords:** dry eye, CFTR, tear fluid

## Abstract

**Purpose:**

Pharmacological activation of ocular surface cystic fibrosis transmembrane conductance regulator (CFTR) chloride channels is a potential pro-secretory approach to treat dry eye disorders. We previously reported the discovery of aminophenyl-1,3,5-triazines, one of which, N-methyl-N-phenyl-6-(2,2,3,3-tetrafluoropropoxy)-1,3,5-triazine-2,4-diamine (herein called CFTR_act_-K267), fully activated human wildtype CFTR with EC_50_ ∼ 30 nM and increased tear volume for 8 hours in mice. Here, functional and pharmacological studies of CFTR_act_-K267 were done in adult New Zealand white rabbits.

**Methods:**

CFTR chloride conductance was measured in vivo by ocular surface potential differences and in ex vivo conjunctiva by short-circuit current. Tear volume was measured by the Schirmer tear test II and CFTR_act_-K267 pharmacokinetics and tissue distribution by liquid chromatography/mass spectrometry. Toxicity profile was studied for 28 days with twice-daily topical administration.

**Results:**

Electrophysiological measurements in vivo and in ex vivo conjunctiva demonstrated CFTR activation by CFTR_act_-K267. A single topical dose of 3 nmol CFTR_act_-K267 increased tear production by >5 mm for 9 hours by the Schirmer tear test, with predicted therapeutic concentrations maintained in tear fluid. No tachyphylaxis was seen following 28-day twice-daily administration, and changes were not observed in corneal surface integrity or thickness, intraocular pressure, or ocular histology. At day 28, CFTR_act_-K267 was concentrated in the cornea and conjunctiva and was not detectable in blood or peripheral organs.

**Conclusions:**

These studies support the development of CFTR_act_-K267 as a pro-secretory therapy for dry eye disorders.

Dry eye disorders constitute a significant health care burden, particularly in an aging population. Current treatment options include artificial tears, punctal plugs, and the topical anti-inflammatory drugs cyclosporine and lifitegrast.^1–3^ There is compelling rationale for development of pro-secretory therapy in dry eye, as increasing the volume of tear fluid bathing the ocular surface is predicted to reduce tear fluid hyperosmolality, which drives the downstream inflammatory response and consequent symptoms.

An attractive target for pro-secretory therapy of dry eye is cystic fibrosis transmembrane conductance regulator (CFTR), a cAMP-regulated chloride channel that is expressed in corneal and conjunctival epithelial cells as well as in various secretory epithelia outside of the eye.^4–11^ Although CFTR at the ocular surface is largely inactive under normal conditions, as it is in the intestine, once activated it can drive fluid secretion at the ocular surface, as it does in the intestine in secretory diarrheas such as cholera.^12,13^ Few ocular surface abnormalities have been reported in cystic fibrosis humans with loss-of-function mutations in CFTR,^14,15^ providing evidence for minimal basal CFTR activity at the ocular surface. As CFTR activation drives fluid secretion by epithelial cells lining the ocular surface, augmentation of tear fluid does not require functional lacrimal or Meibomian glands.

We recently identified by high-throughput screening an aminophenyl-1,3,5-triazine class of small molecule activators of wildtype CFTR. A compound from the screen, CFTR_act_-K089 (Fig. 1), fully activated CFTR in cell cultures with EC_50_ ∼250 nM and, when delivered topically to mice, doubled tear volume for 4 hours.^16^ In lacrimal gland ablation models in mice, CFTR_act_-K089 administered three times daily normalized tear volume, prevented corneal epithelial disruption, and even reversed pathology when administered after development of dry eye. In a recent medicinal chemistry study, the CFTR_act_-K089 analog *N*-methyl-*N*-phenyl-6-(2,2,3,3-tetrafluoropropoxy)-1,3,5-triazine-2,4-diamine (herein called CFTR_act_-K267) fully activated CFTR with EC_50_ ∼ 30 nM and produced a sustained increase in tear volume in mice for 8 hours following 25 pmol topical administration.^17^ CFTR_act_-K267 was without effect in CFTR-deficient mice and was rapidly metabolized by the liver, a desirable characteristic for minimizing systemic exposure.

**Figure 1:**
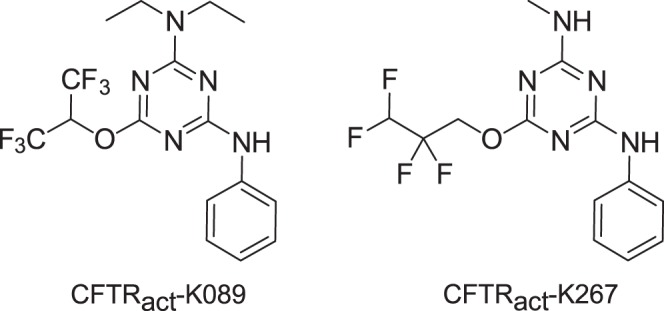
Chemical structures of CFTR activators CFTR_act_-K089 and CFTR_act_-K267.

Here, to advance CFTR_act_-K267 in its preclinical development, the activity and pharmacology of CFTR_act_-K267 were studied in rabbit as a model of human ocular surface physiology.

## Materials and Methods

### Rabbits

A total of 24 adult female New Zealand white rabbits (Western Oregon Rabbit Co., Philomath, OR, USA) weighing 2 to 3 kg were used for this study. Rabbits were acclimated for 3 days prior to experiments and raised under standard laboratory conditions. Rabbit protocols were approved by the University of California, San Francisco Institutional Animal Care and Use Committee and conducted in accordance with the ARVO Statement for the Use of Animals in Ophthalmic and Vision Research.

### Chemicals and Formulation

CFTR_act_-K267 was synthesized by stepwise substitution reactions of cyanuric chloride with methylamine, 2,2,3,3-tetrafluoropropanol, and aniline under basic conditions, as described,^17^ and purified to >95% by flash chromatography (1:2 ethyl acetate:hexane). CFTR_act_-K267 was prepared as a 10 mM dimethyl sulfoxide (DMSO) stock solution. The ophthalmic formulation contained 0.22-μm filtered Ringer's solution containing 0.3% carboxymethylcellulose (CMC, high viscosity; VWR, Radnor, PA, USA), 0.015% benzalkonium chloride preservative, and 1% DMSO at pH 7.40. For some studies a higher concentration of CMC (0.665%) was used to increase viscosity.

### Ocular Surface Potential Difference Measurements

**Figure 2:**
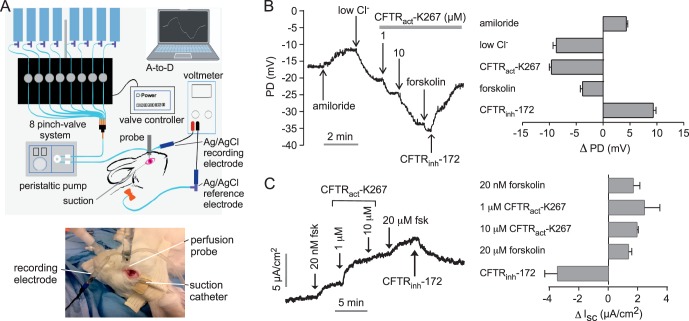
Electrophysiological analysis of CFTR activation by CFTR_act_-K267 at the rabbit ocular surface. (A) Schematic (top) and photograph (bottom) of ocular surface potential difference (PD) recording method. The perfusion catheter coupled to the measuring electrode was oriented perpendicular to the ocular surface near the medial canthus. The eyelids create a natural reservoir for corneal and conjunctival exposure, with vacuum aspiration maintaining a stable perfusate volume. (B) Left: Representative ocular surface PD recording in response to sequential solution exchanges. Right: Summary of PD changes (Δ PD) in response to indicated maneuvers (mean ± SEM; n = 16 eyes). (C) Left: Representative short-circuit current (Isc) measurement in freshly isolated rabbit forniceal and palpebral conjunctiva in response to compound additions. Right: Summary of changes in Isc (Δ Isc) in response to compound additions (mean ± SEM; n = 3).

Open-circuit transepithelial potential difference (PD, in mV) at the ocular surface was measured continuously in anesthetized rabbits using a procedure modified from that established in mice.^8^ Rabbits were intubated and anesthetized with isoflurane, and respiratory rate, blood pressure, and body temperature were monitored. For PD recording, solutions (see below) were serially perfused at 10 mL/min through PE-90 plastic tubing using a gravity multireservoir pinch-valve system (ALA Scientific, Westbury, NY, USA) and a variable-flow peristaltic pump (medium flow model; Thermo Fisher Scientific, Fair Lawn, NJ, USA). A perfusion catheter was fixed onto an adjustable stereotaxic frame with the tip immersed in solution contacting the ocular surface, as diagrammed in Figure 2A. Excess fluid was aspirated by continuous suction (low-powered wall vacuum) using 1/8-inch tubing (inner diameter 3/32 inch) placed 3 mm from the lateral canthus to maintain near-constant perfusate volume in contact with cornea, bulbar conjunctiva, and palpebral conjunctiva without fluid runoff. The measuring electrode contacted the perfusion catheter and was connected to a high-impedance voltmeter (IsoMilivolt Meter; World Precision Instruments, Sarasota, FL, USA). The reference electrode was grounded using a winged, 25-gauge needle filled with normal saline inserted subcutaneously in the abdomen. The measuring and reference electrodes consisted of Ag/AgCl with 3 M KCl agar bridges.

Solutions consisted of the following: (1) normal Cl^−^ solution (mM): 99 NaCl, 24 KCl, 32 NaHCO_3_, 1.0 NaH_2_PO_4_, 0.6 MgCl_2_, 0.8 CaCl_2_; (2) normal Cl^−^ solution with amiloride (100 μM); (3) low Cl^−^ solution with amiloride (NaCl replaced by Na gluconate and KCl by K gluconate); (4) low Cl^−^ + amiloride + CFTR_act_-K267 (1 μM); (5) low Cl^−^ + amiloride + CFTR_act_-K267 (10 μM); (6) low Cl^−^ + amiloride + CFTR_act_-K267 (10 μM) + forskolin (20 μM); (7) low Cl^−^ + amiloride + forskolin + CFTR_act_-K267 (10 μM) + CFTR_inh_-172 (10 μM). The solutions were isosmolar to rabbit tear film (302 mOsm) with a pH of 7.4. All solutions contained 10 μM indomethacin to prevent CFTR activation by prostaglandins. As has been done in many tissue electrophysiology studies, indomethacin was included to minimize stress- or trauma-induced prostaglandin production. Indomethacin was found to improve reproducibility in our previous mouse ocular PD studies.^8^

### Short-Circuit Current Measurements

Short-circuit current was measured in freshly isolated rabbit conjunctiva, as described^16^ with modification. Rabbits were euthanized by injection of 150 mg/kg euthasol into the marginal ear vein. The entire eyeball with eyelids intact was removed from the orbit to preserve conjunctival epithelial integrity. Within 10 minutes, a sheet of forniceal and palpebral conjunctiva was dissected, mounted onto a P2304 tissue slider (Physiologic Instruments, San Diego, CA, USA), and placed into the Ussing chamber. The apical and basolateral chambers contained (in mM): 120 NaCl, 3 KCl, 1 CaCl_2_, 1 MgCl_2_, 10 glucose, 25 NaHCO_3_, and 5 HEPES, pH 7.4. Solutions were bubbled with 95% O_2_/5% CO_2_ and maintained at 37°C. Hemichambers were connected to a DVC-1000 voltage clamp (World Precision Instruments Inc., Sarasota, FL, USA) via Ag/AgCl electrodes and 3 M KCl agar bridges.

### Schirmer Tear Test II

Tear production was measured using the anesthetized Schirmer Tear Test (STT, Eagle Vision, Memphis, TN, USA). To minimize reflex tearing, one drop of 0.5% proparacaine hydrochloride (Akorn, Lake Forest, IL, USA) was placed onto the corneal surface and excess fluid was absorbed at the medial canthus using eye spear sponges (Fine Science Tools, Foster City, CA, USA). After 5 minutes, the notched strip was inserted into the lower lateral conjunctival fornix, maintaining contact with the lateral cornea. The wetted length (mm) of the strip indicated by blue dye appearance was read after 5 minutes.

### Pharmacodynamics

Serial STT measurements were done before and at 1, 3, 6, 9, 12, and 24 hours following application of a single 30-μL drop of CFTR_act_-K267 formulation (or vehicle alone) into the conjunctival sac. Both eyes received the same treatment to control for potential contralateral effects of a given treatment.

### Pharmacokinetics and Tissue Distribution

CFTR_act_-K267 was quantified by liquid chromatography/mass spectroscopy (LC/MS) at 15 minutes, 1 hour, 2 hours, 6 hours, and 24 hours after a single, 3-nmol topical dose. To recover CFTR_act_-K267 at indicated times, three eye washes of 30 μL sterile PBS were done with solution recovered from the lateral and medial canthi after manual eyelid blinking using 50-μL glass capillary tubes. Pooled washes were diluted in three volumes of ethyl acetate, centrifuged at 18,500*g* for 15 min, and the collected supernatant was analyzed by LC/MS (Waters 2695 HPLC with Micromass ZQ).^16^ LC was done on an Xterra MS C18 column (2.1 mm × 100 mm, 3.5 μm) with 0.2 mL/min water/acetonitrile containing 0.1% formic acid, 12-minute linear gradient, and 5% to 95% acetonitrile.

In chronic treatment studies, CFTR_act_-K267 (3 nmol, or formulation control) was given twice daily (8 AM and 4 PM) for 28 days. Two mL of blood was collected from the marginal ear vein in EDTA tubes. Rabbits were then euthanized using 150 mg/kg euthasol, and ocular tissues, blood, and peripheral organs were collected. Using a surgical microscope, 150 μL of aqueous humor was collected through the peripheral cornea using a 25-gauge needle, and 300 μL of vitreous fluid was aspirated through the pars plana with a 23-gauge needle. Following transcardial perfusion with heparinized PBS, the eyes were enucleated with lids intact, and the cornea, iris/ciliary body, lens, bulbar, forniceal and palpebral conjunctiva, and retina of both eyes were dissected, weighed, homogenized in a 1:4 mixture of water:ethyl acetate (10 mL/1 g tissue), and centrifuged (1000*g* for 15 minutes). Plasma, aqueous, and vitreous samples were each mixed with 3 volumes of ethyl acetate and centrifuged for 15 minutes at 18,500*g*, and the supernatant was evaporated and redissolved in HPLC eluent (100 μL of 1:3 water:acetonitrile containing 0.1% formic acid) for LC/MS analysis. Also, the brain, kidney, heart, and liver were removed, weighed, mixed in a 1:4 mixture of water:ethyl acetate (10 mL/1g tissue), and homogenized. The homogenized samples were vortexed and centrifuged (1000*g* for 15 minutes), and the ethyl acetate-containing supernatant was evaporated and then redissolved in HPLC eluent for LC/MS analysis. The lower limit of detection for CFTR_act_-K267 was ∼0.2 pg/mg of homogenized tissue or biological fluid, which was defined as giving a signal-to-noise ratio >3.

### Clinical Examination

In chronic treatment studies, eyes were treated twice daily for 28 days as described previously. STT, intraocular pressure (IOP), and central corneal thickness were measured on days 0, 7, 14, 21, and 28. Slit lamp examinations were performed on days 0, 14, and 28 by a board-certified ophthalmologist blinded to treatment status. STT was done 1 hour after the first treatment of the day (9 AM). IOP was measured with a Tonolab rebound tonometer (Colonial Medical Supply, Windham, NH, USA). Central corneal thickness was measured using the Corneo-Gage Plus 2 pachymeter (Sonogage Inc., Cleveland, OH, USA). For slit lamp examination, Lissamine green strips (GreenGlo, HUB Pharmaceuticals LLC, Rancho Cucamonga, CA, USA) were wetted with 25 μL lubricant eye drops and then applied gently into the inferior fornix. One minute later, photographs of the eye were taken with a digital camera and staining was evaluated according to a 12-point scale as described^18^: each corneal quadrant was scored in a blinded fashion on a 3-point scale: grade 0, no staining; grade 1, sporadic staining (involving <25% of the total surface); grade 2, diffuse punctate staining (25%–75%); and grade 3, coalesced punctate staining (≥75%). The total grade is reported as the sum of scores from all four quadrants, ranging from 0 to 12. Conjunctival congestion, chemosis, discharge, corneal haze or neovascularization, anterior chamber cellular reaction or flare, iris neovascularization, lens opacification, or loss of red reflex were each rated using a modified four-point McDonald-Shadduck scale, where zero is normal.

### Histology

A subset of chronically treated eyes were enucleated with eyelids intact after transcardial perfusion with PBS followed by 4% paraformaldehyde and left overnight in 4°C in 30% sucrose. Eyes were embedded in optimal cutting temperature compound (OCT) and sectioned through central cornea, posterior pole, and superior and inferior fornices/eyelids. Cryosections (8 μm thickness) were stained with hematoxylin and eosin using a standard protocol.

### Statistics

Data are presented as mean ± SEM. Statistical analyses were performed using GraphPad Prism software (GraphPad, San Diego, CA, USA). Serial tear volume measurements, IOP, and corneal thickness were analyzed by 2-way ANOVA with Dunnett post hoc analysis.

## Results

### CFTR_act_-K267 Activates CFTR Chloride Conductance at the Rabbit Ocular Surface

CFTR_act_-K267 activity at the ocular surface in rabbits in vivo was measured using an open-circuit PD method, as developed originally in mice. The method involves perfusion of the ocular surface with a series of solutions during continuous measurement of PD using a high-impedance voltmeter, as diagrammed in Figure 2A. The average absolute PD measured initially was −14 ± 1 mV (mean ± SEM, *n* = 16 eyes). The representative PD curve in Figure 2B (left) shows an initial depolarization following addition of the ENaC inhibitor amiloride, with hyperpolarizations following perfusion with low Cl^−^ solutions without and then with CFTR_act_-K267, and then with a high concentration of the cAMP agonist forskolin to maximally activate CFTR. The CFTR inhibitor CFTR_inh_-172 was present in the final perfusion solution. CFTR_act_-K267 produced a substantial depolarization that was minimally further increased by forskolin, with the depolarizations largely reversed by CFTR_inh_-172. Averaged changes in PD from measurements done on 16 eyes are summarized in Figure 2B (right). These results confirm activation of CFTR at the rabbit ocular surface by CFTR_act_-K267. However, ocular-surface PD data should be considered semiquantitative because of nonlinearity in PD values with CFTR function and because of uncertainties in the extent of perfusate fluid contact with whole ocular surface and of compound accumulation in ocular surface cells.

In separate electrophysiological studies, CFTR activation was measured in freshly isolated conjunctiva ex vivo by short-circuit current analysis. The representative curve in Figure 2C (left) shows a small increase in current in response to addition of a low concentration of forskolin (25 nM), which was further increased by 1 and then 10 μM CFTR_act_-K267. Maximal CFTR activation was produced by a high concentration of forskolin. The increases in short-circuit current were inhibited by CFTR_inh_-172. Amiloride (10 μM) had no effect on short-circuit current (not shown). Averaged changes in short-circuit current are summarized in Figure 2C (right). These ex vivo results confirm CFTR_act_-K267 activation of CFTR in conjunctival epithelium, with the data at 1 versus 10 μM CFTR_act_-K267 indicating an apparent EC_50_ < 1 μM.

### CFTR_act_-K267 Pharmacodynamics

**Figure 3:**
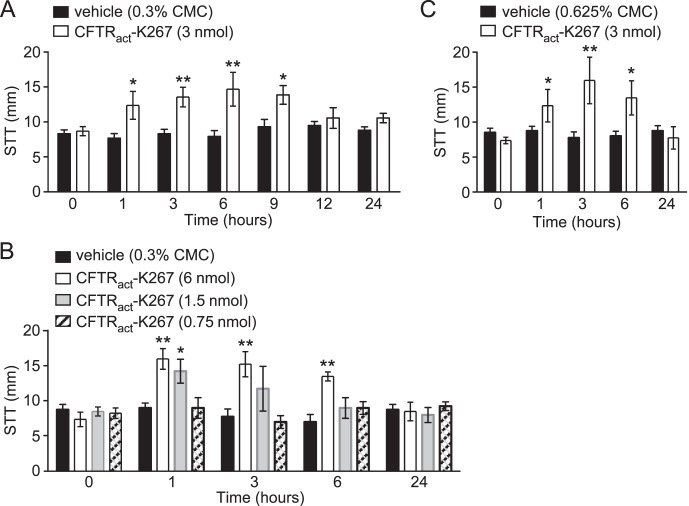
CFTR_act_-K267 increases tear fluid at the rabbit ocular surface as measured by Schirmer's test. (A) Tear volume (mm, by Schirmer's test) measured just before and at the indicated times after single-dose topical application of 3 nmol CFTR_act_-K267 or formulation (containing 0.3% CMC) control (mean ± SEM; 8 eyes per condition). (B) Dose dependence with study done as in A, comparing 0.75, 1.5, and 6.0 nmol CFTR_act_-K267 (4 eyes per condition). (C) Effect of formulation viscosity, with study done as in A, for formulation containing 0.665% CMC instead of 0.3% CMC (4 eyes per condition). *P < 0.05, **P < 0.01, ANOVA, comparing CFTR_act_-K267 versus vehicle-treated eyes.

CFTR_act_-K267 was tested for its efficacy in augmenting tear fluid production in rabbits. Prior work identified a formulation (0.325% CMC in Ringer's solution containing 0.015% benzalkonium chloride and 1% DMSO) that stably solubilized CFTR_act_-K267 and was effective when delivered topically to mice.^17^ A single application of 3 nmol CFTR_act_-K267 (10 μL of a 100 μM solution) increased tear volume by ∼60% for at least 9 hours when compared with vehicle (Fig. 3A). Dose-dependence studies showed similar activity of 6 nmol CFTR_act_-K267, but reduced duration of activity with 1.5 nmol CFTR_act_-K267 and no significant increase in tear production with 0.75 nmol CFTR_act_-K267 (Fig. 3B). When 3 nmol of CFTR_act_-K267 was delivered in a more viscous formulation containing 0.625% (instead of 0.3%) CMC, to potentially increase CFTR_act_-K267 ocular surface residence time, compound efficacy was unchanged (Fig. 3C).

### CFTR_act_-K267 Pharmacokinetics

**Figure 4:**
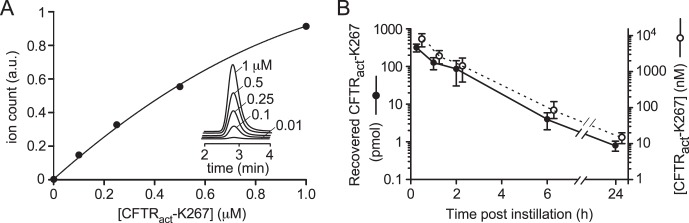
CFTR_act_-K267 concentration in rabbit tear fluid following instillation of a single 3-nmol dose. (A) Standard LC/MS curve of aqueous solutions containing specified concentrations of CFTR_act_-K267. (B) Recovered CFTR_act_-K267 (in pmol, closed circles, left ordinate) and deduced concentration (in nM, open circles, right ordinate) in tear fluid. Each point is the average of measurements done on 2 eyes for each time point.

Pharmacokinetics in tear fluid was measured by LC/MS analysis of material recovered in three eye washes done at specified times following a single topical dose of 3 nmol CFTR_act_-K267. Figure 4A shows original LC/MS data and a standard curve from which the amount of recovered CFTR_act_-K267 was deduced. Figure 4B shows an approximate exponential decline in CFTR_act_-K267 recovered from tear fluid (closed circles, left axis). Corresponding compound concentrations in tear fluid (open circles, right ordinate) were estimated using tear volumes deduced from STT measurements in Figure 2A. These results support the conclusion that CFTR_act_-K267 remains at predicted therapeutic concentrations in tear fluid for at least several hours following administration of a 3-nmol dose.

### Chronic Administration Studies

**Figure 5:**
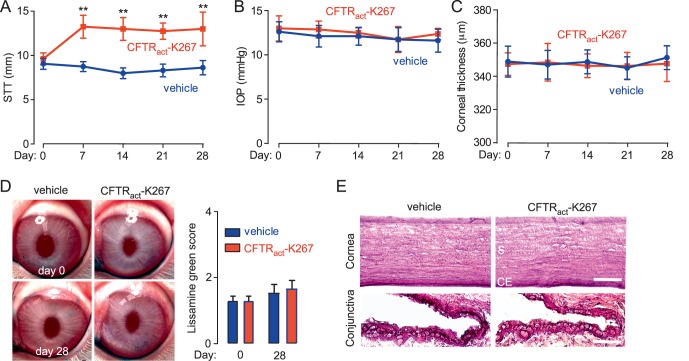
Ocular toxicity studies in a chronic CFTR_act_-K267 administration model. Rabbits were treated with 3 nmol CFTR_act_-K267 (or vehicle control) twice daily for 28 days. STT (A), IOP (B), and central corneal thickness (C) before and weekly following initiation of CFTR_act_-K267 administration (mean ± SEM, 8 eyes). **P < 0.01, ANOVA, comparing CFTR_act_-K267 versus vehicle-treated eyes. (D) Left: Representative photographs taken before and at day 28. Right: Lissamine green staining scores (mean ± SEM, 8 eyes). (E) Hematoxylin and eosin staining of the cornea and the conjunctiva at day 28, representative of sections done on 2 eyes per group. S, stroma; CE, corneal endothelium. Scale bars: 100 μm (cornea), 25 μm (conjunctiva).

Repeated topical delivery of CFTR_act_-K267 (3 nmol, twice daily for 28 days) augmented tear volume in a sustained fashion without tachyphylaxis (Fig. 5A). No significant differences were found comparing vehicle and CFTR_act_-K267-treated eyes on IOP (Fig. 5B) or central corneal thickness (Fig. 5C). No apparent acute ocular irritation was observed following topical administrations, as evidenced by a lack of excessive blinking or altered behavior. Slit-lamp evaluation showed no evidence of conjunctival hyperemia, anterior chamber inflammation, or lens opacification. Lissamine green staining showed no injury to the ocular surface in vehicle-treated and CFTR_act_-K267-treated eyes (Fig. 5D). Histology showed no pathological changes in the cornea or conjunctiva at day 28 (Fig. 5E) or in the lens, ciliary body, or retina (not shown).

**Figure 6:**
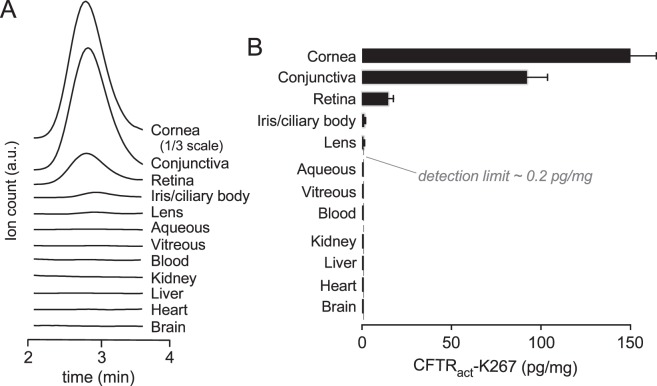
Tissue levels following chronic CFTR_act_-K267 administration (3 nmol twice daily for 28 days). (A) Representative LC/MS elution curves shown for CFTR_act_-K267 in indicated tissues. (B) CFTR_act_-K267 levels in ocular and extraocular tissues (mean ± SEM, 8 rabbits). LC/MS detection limit shown as vertical dashed line.

Following the chronic treatment, CFTR_act_-K267 was below the limit of detection by LC/MS in the blood, heart, brain, liver, and kidney (Figs. 6A, 6B), indicating minimal systemic exposure, as expected, given the rapid predicted hepatic metabolism of CFTR_act_-K267 deduced from in vitro microsomal stability measurements.^17^ In ocular tissues, the LC/MS analysis showed CFTR_act_-K267 in cornea > conjunctiva >> retina, with levels near or below the limit of detection in the aqueous and vitreous fluid, lens, and iris/ciliary body. The low but measurable level in retina, equivalent to <10 nM CFTR_act_-K267, may result from trans-scleral transport because CFTR_act_-K267 was not detected in the vitreous fluid.

## Discussion

The functional data showed rapid and prolonged activation of CFTR chloride channels at the rabbit ocular surface following exposure to CFTR_act_-K267. A single topical dose of 3 nmol CFTR_act_-K267 produced a substantial and sustained increase in tear secretion for at least 9 hours, which, if translated to human dry eye, could have therapeutic efficacy with once- or twice-daily dosing. The sustained augmentation in tear production over 9 hours, averaging 5.3 mm by STT, corresponds to a 2.7 μL increase in tear fluid volume using the reported relationship between STT wetting and volume.^19,20^ This study in rabbits builds on our prior studies in mice,^16,17^ as rabbits are suitable for detailed pharmacokinetic, tissue distribution, and pharmacodynamic measurements as well as long-term toxicity studies with noninvasive serial assessments of corneal health and intraocular pressure. Also, rabbit eyes are considered better surrogates for human eyes than are rodent eyes.

Prior studies showed CFTR activation by CFTR_act_-K267 with nanomolar potency and without detectable elevation of total cellular cAMP.^17^ The absence of CFTR_act_-K267 effect in CFTR-deficient mice supported CFTR-dependent action at the ocular surface. Pharmacodynamic and pharmacokinetic studies here in rabbits showed sustained elevation in tear aqueous production with predicted therapeutic concentrations of CFTR_act_-K267 in tear fluid. Chronic administration studies with twice-daily dosing for 28 days revealed CFTR_act_-K267 accumulation in ocular tissue, mainly in the cornea > conjunctiva >> retina, with levels below the limit of detection in the aqueous and vitreous fluid and in the blood and peripheral tissues. Ocular toxicity was not observed as assessed by in vivo examination of the ocular surface, cornea, and lens, by measurements of intraocular pressure and corneal thickness, and by ocular histology. Together these finding support the development of CFTR_act_-K267 for dry eye disorders.

The potential difference measurement method used here, which was developed initially for studies of ion channels at the mouse ocular surface,^8,21^ was motivated by nasal potential difference measurements used for decades to study CFTR function in cystic fibrosis.^22^ Unlike short-circuit current measurements in isolated cornea or conjunctiva,^18,23^ ocular surface PD measurements provide information about CFTR function in its native environment in which ocular surface anatomy and physiology are preserved, which is important because of heterogeneity in transport properties of the cornea and conjunctiva^21,24–26^ and because of possible changes in basal cyclic nucleotide levels following tissue excision. The PD results here in rabbits showed rapid CFTR activation at the ocular surface with maximal effects within a few minutes after exposure to CFTR_act_-K267. Because of its simplicity and good signal to noise, measurements of ocular surface PD may be translatable to humans as a surrogate functional assay of drug candidates targeting ion channels.

The PD results showed that amiloride produced a depolarization averaging ∼5 mV, much smaller than the total hyperpolarization of >20 mV. Albeit a small depolarization, this finding implicates the presence of an amiloride-sensitive epithelial sodium channel, presumably ENaC, somewhere on the ocular surface. However, no amiloride effect was seen in short-circuit measurements on isolated conjunctiva, which might be the consequence of the particular area of conjunctiva studies or the limited sensitivity of the method. Prior studies on sodium channels at the rabbit ocular surface, largely in the older literature, reported mixed findings. Evidence for an amiloride-insensitive sodium pathway was reported in rabbit cornea^26,27^ and conjunctiva.^28,29^ Other studies, however, have reported some amiloride-sensitive ENaC activity at the rabbit ocular surface in vivo.^30,31^ It will be important to establish the presence and magnitude of amiloride-inhibitable ENaC activity at the human ocular surface for consideration of ENaC inhibitor therapy for dry eye.

The substantial and sustained CFTR activation at the ocular surface produced by CFTR_act_-K267, without tachyphylaxis, is consistent with the known biology of CFTR as studied extensively in the airways and intestine.^10,32,33^ An alternative pro-secretory strategy for dry eye is pharmacological activation of calcium-activated chloride channels, which are thought to be expressed on conjunctival epithelia, mucin cells, and lacrimal glands.^23,34^ The UTP analog diquafosol, which activates epithelial P2Y_2_ receptors and downstream calcium signaling, has been approved for dry eye in Japan, but did not show efficacy in phase III trials in the United States,^35^ perhaps because of the only transient calcium elevation and consequent chloride channel activation produced by P2Y_2_ agonists.

Another pro-secretory strategy for increasing tear fluid is anti-absorptive therapy by inhibition of ENaC sodium channels. In a phase I/IIa study, the ENaC inhibitor P321 has shown tolerability and safety in patients with mild to moderate dry eye (Boyer JL, et al. *IOVS* 2016;57:ARVO E-Abstract 2875), and a phase II study is in progress. Theoretical modeling supports the efficacy of an anti-absorptive approach to increase tear fluid, albeit with lower efficacy than a pro-secretory approach; modeling also supports the additive action of anti-absorptive and pro-secretory drugs.^21^ We note that pro-secretory or anti-absorption drugs are combinable with anti-inflammatory drugs because they target distinct mechanisms in dry eye pathogenesis. Finally, we note that pro-secretory or anti-absorptive therapy may not correct lipid or mucin deficiency in some cases of dry eye; however, augmentation of aqueous volume is predicted to correct tear fluid hyperosmolality and downstream inflammation even in evaporative dry eye.

Although the results here support the development of CFTR_act_-K267 for testing in human dry eye, several limitations are noted. Optimization of the ocular formulation to deliver CFTR_act_-K267 is needed, as the studies here used an original formation established previously^17^ that contained DMSO to ensure compound solubility. Moreover, longer term and systemic toxicity studies, plus evaluation in additional species, may be needed for further preclinical development. In addition to STT measurement for tear production, other measures of efficacy might include tear break-up time and tear fluid osmolality. Finally, although rabbit eyes are more similar to human than to rodent eyes, they may differ from human eyes in their capacity to produce lacrimal fluid, in their corneal epithelial thickness, and in their tolerability to ocular irritation, and it is not known whether the rabbit nictitating membrane contains conjunctival epithelium expressing CFTR that may contribute to ocular fluid secretion.^36^

## Conclusions

In summary, a small molecule CFTR activator with nanomolar potency was effective in producing sustained tear fluid hypersecretion in rabbits following single-dose topical administration. At therapeutic doses administered twice daily for 28 days, compound activity was not diminished, no signs of ocular toxicity were observed, and the compound was not detectable outside of the eye. CFTR_act_-K267 may thus be a safe and effective therapy for human dry eye disorders alone or when combined with other dry eye medications.
